# Resilience and Poly-Victimization among Two Cohorts of Norwegian Youth

**DOI:** 10.3390/ijerph15122852

**Published:** 2018-12-13

**Authors:** Lihong Huang, Svein Mossige

**Affiliations:** 1 Youth Research, NOVA, Oslo Metropolitan University, PB 4 St. Olavs plass, N-0130 Oslo, Norway; 2 Department of Psychology, University of Oslo, PB 1072 Blindern, N-0316 Oslo, Norway; sveinmos@psykologi.uio.no

**Keywords:** resilience, mental health, poly-victimization, sexual abuse, physical violence, verbal bullying

## Abstract

Previous research shows that there is a significant and positive relationship between being a victim of violence and experiencing high levels of psychological problems among young people. Conversely, resilience is negatively associated with psychological problems among young people in general, and this negative association is particularly strong among victims of violence. Our study examines resilience among young people (aged ≥ 18 years) who reported being victims of multiple forms of violence during childhood and adolescence using data from two national youth surveys administered in Norway in 2007 (*N* = 7033) and 2015 (*N* = 4531), respectively. We first compared the score of resilience, as measured by the Resilience Scale for Adolescents (READ), and the prevalence of poly-victimization, as identified by the number of young people in our study who were exposed to three of the four forms of violence (i.e., non-physical violence, witnessing violence against parents, physical violence, and sexual abuse). Second, we tested our hypothesis using our data and found that resilience—individuals’ capacity to handle adversity, as well as their use of social and cultural resources when facing adversity—moderates the association between poly-victimization and the onset of psychological problems.

## 1. Introduction

Over the past decade, there has been a rapid development in the various concepts and methodological approaches used to estimate the prevalence of children and adolescents who are victims of violence. Musicaro et al. [1] clarify the terminology associated with recurrent interpersonal victimization; the authors expressed how cumulative trauma theory emphasizes the relationship between the frequency and severity of victimization, and they posited a linear association between the number of traumatic event types and the severity of clinical impairment. Two terms have been used to describe children and youth who have been exposed to several types of violence: poly-victimization [2] and multiple victimization [3,4]. The term ‘multiple victimization’ is also referred to as ‘multi-type maltreatment’ [5] or ‘multiple maltreatment’ [6], and it indicates that different victimization types tend to co-occur [7], or that multiple types of victimization occur across multiple areas, such as with peers, family, and in the community [8]. Although the term is relatively new and still under debate among scholars, the term ‘poly-victimization’ was found to be suitable in cross-sectional studies due to its emphasis on ‘cumulative trauma’ (i.e., different forms and types of violence, rather than cumulative episodes of the same kind of victimization) [9].

Poly-victimization, by definition, necessitates that assessments used to measure its presence and severity should include different kinds of victimization, rather than multiple episodes of the same kind of victimization [10]. In a few prevalence studies of poly-victimization using representative samples, such as those conducted in the United Kingdom [11], Spain [12], Canada [13], Sweden [14], and Norway [9], there is a lack of consensus in terms of the measurement of poly-victimization and the cutoff point that determines who can qualify as a poly-victim. Nevertheless, previous research findings have led to the claim that there is a strong, linear relationship between poly-victimization and mental health over both the short term and long term, especially among children and adolescents. Moreover, the accumulation of poly-victimization during childhood may have significant negative effects on children’s self-regulatory development [15] and can result in the development of mental health disorders later in life [16].

Most of the previous research on poly-victimization has focused on the risk factors associated with the development of mental health disorders [17] and little has delved into the factors of resilience that may moderate these negative effects. In recent years, resilience has received considerable attention as a desirable behavioral adaptation when one faces adverse experiences [18]. Resilience is partly understood as a personal trait inherent in an individual, and it is partly regarded as a process or phenomenon influenced by culture and context [19]. This concept is particularly interesting and relevant for children and adolescents who are victims of violence, as resilience represents individuals’ capacity to navigate their way to health-sustaining resources. Resilience is also dependent on the individual’s family, community, and culture that provide these health resources and experiences in the face of adversity [20]. Meanwhile, significant gender differences are found when examining one’s exposure to different forms of violence [21,22]. Gender, together with its intersection and interaction with migration, experience of violence, and resilience, is a well-established predictor of mental health among both adolescents and adults [23,24].

This study has three aims: The first is to identify whether there is a difference in the prevalence of poly-victimization reported by Norwegian youth between two cohorts (2007 and 2015). The second is to test the association between poly-victimization and mental health. The third is to examine whether resilience may moderate the negative effects of poly-victimization on adolescents’ mental health. Using data from two national youth surveys conducted in Norway in 2007 and 2015, we can identify whether there is a stable trend in the prevalence of poly-victimization and can examine its negative effects on mental health among Norwegian adolescents. In addition, we can assess whether resilience moderates the negative relationship between poly-victimization and mental health.

## 2. Materials and Methods

### 2.1. Procedure

The data used in this study were obtained from the Norwegian youth survey on violence and abuse, which was first administered in 2007 and then again in 2015 [25]. The aim of the surveys was to assess the prevalence of four different offences against children and youth: non-physical violence, witnessing violence at home, physical violence, and sexual abuse. The 2007 survey used a stratified random sample of 67 upper-secondary schools. To obtain a nationally representative sample, Statistics Norway included every school in the country in a pool from which the participating schools were selected. The sampling procedure included three steps: first, the country was stratified into five geographic regions; second, all upper-secondary schools in a region were stratified into three groups according to the type of education offered in the schools, such as general study, vocational study, and a combination of the two. These two steps resulted in 15 strata. Then, as the third step, one school from each stratum was randomly selected to the school sample. The 2015 survey used the same sample of upper-secondary schools that participated in the 2007 survey. Five schools had either closed or merged with other schools. Of the remaining 62 schools, 41 agreed to participate. Since the high schools in Norway substantially increased in size from the years 2007 to 2015, eight additional schools were invited to participate as replacements to obtain the desired sample size, resulting in a total sample of 49 schools. The replacement schools were selected from the same strata of schools that had either closed down or refused to participate. All students in their last year of education at these schools were invited to participate in the survey. The choice to include only those students in their last year of upper-secondary school to complete the surveys was based on the certainty that the students were all aged 18 years and older, and they were able to provide their own informed consent to participate in the study. According to the Norwegian Data Protection Services (NSD) regulations, children aged 15 years and older can provide their own informed consent to participate in research that involves completing an ordinary survey, but they have to be older than 16 years old to provide their own consent to participate in a survey that collects sensitive personal information, such as data on their sexual or political orientations.

Complying with §33 and §34 of the Personal Data Act (Act of 14 April 2000 No. 31 relating to the processing of personal data), both surveys were approved by the NSD (Ref.14/01407-5/EOL) prior to data collection. We then contacted the schools participating in our study to obtain permission at the school level to administer the survey. Students at each school provided their own informed consent to participate in the study after receiving written information that clearly identified the themes and purpose of the study. The written information provided to the schools and to individual students emphasized that participation in the study was voluntary and all participants’ answers would remain anonymous. The anonymity of the survey was further safeguarded at the school level and at the individual level, as we did not request any personally identifying data. During both data-collection periods, all students were invited to complete a questionnaire on an individual computer during school hours with a teacher present. As the topics in the questionnaire were sensitive and could possibly trigger unpleasant memories or feelings in the respondents, we provided information about where and with whom (i.e., a counselor or health care professional at school) the respondents could seek help; this information was presented both at the start of the survey and on the last page of the survey questionnaire.

Data are available at Voldprogrammet–Forskningsprogram on vold i nære relasjoner (in English: The Domestic Violence Research Program–A research program about violence in close relationships; https://blogg.hioa.no/voldsprogrammet/forskere/). Since Norway is a small country, these survey data must be stripped of several background variables before being made fully accessible without restriction. Therefore, as per our usual practice, data are safely stored and accessible from the NOVA–OsloMet Institutional data Access/Ethics Committee, which will grant researchers access to the data if they meet the criteria to access confidential data.

### 2.2. Participants

Overall, 7033 students responded to the 2007 survey, with a response rate of 77.3%; conversely, 4530 students responded to the 2015 survey, which provided a sufficient response rate of 66.2%. After conducting some preliminary data analyses, we did not observe any systematically missing data from the 2015 survey. Moreover, previous analyses [26] comparing the two samples found no correlations between reports of violence and response rates at the participating schools in both surveys, and there were no significant differences in the proportion of girls, the proportion of students from immigrant backgrounds, nor the proportion of participants who reported that both parents were unemployed. However, there was a significant and slightly higher proportion of participants who reported that their parents had attained higher education levels in the 2015 cohort when compared with the 2007 cohort, which is in line with developments in the general population, as more people have been attaining higher education in Norway over the last decade. In both datasets, nearly all respondents (99.8% in 2007 and 100% in 2015) were 18 years of age or older and 59% of respondents were female. Further, the student population has an equal gender ratio in Norway. All children receive mandatory entrance into upper-secondary education at the age of 16 following 10 years of compulsory education. One factor that may have contributed to the low participation rate of male students is that they may not have been at school during the data-collection periods, as they were mostly completing field practice during their last year of upper-secondary vocational education, which is a male-dominated educational track in Norway.

### 2.3. Measures of Victimization across Four Forms of Violence

Both the 2007 and 2015 surveys obtained data on four major types of offences against children: (1) experiencing non-physical violence (severe verbal bullying, threats of violence) perpetrated by parents and/or peers; (2) being a witness of domestic violence against their parents and siblings; (3) experiencing physical violence (slap with open hands, fists, or being “beaten up”) perpetrated by parents or peers; and (4) experiencing sexual abuse (unwanted sexual events with physical contact). The questions on the questionnaire investigating the four major forms of violence are presented in similar words for both surveys: “Has any of the following ever happened to you?” Table A1 in the Appendix A presents all items used in both surveys that measured all four forms of violence, as well as the items used to assess the respondents’ prevalence of being a victim of violence.

We counted all events where the respondents chose the response options ‘yes’, ‘once’, or ‘more times’ on those items that measured physical and sexual abuse, while excluding items measuring non-physical/verbal abuse that occurred only once; this was in line with a previous definition of repeated bullying [27]. Measures of non-physical abuse included the following sub-categories: (1) verbal bullying and threats of violence perpetrated by peers, and (2) verbal bullying and threats of violence perpetrated by parents. Witness to violence included (1) witnessing verbal abuse between parents, (2) witnessing physical violence between parents, and (3) witnessing violence against siblings perpetrated by parents. Measures of physical violence included (1) being beaten or injured by peers, and (2) physical abuse perpetrated by parents. Measures of sexual abuse included items relating to unwanted sexual events with physical contact.

### 2.4. Measures of Mental Health

We used a 13-item short version and 26-item full version of the Hopkins Symptom Checklist (HSCL) as a proxy measure of mental health, which has been validated in the Norwegian national youth surveys since the 1980s [28]. The questions asked whether various symptoms of depression and anxiety were experienced during the past week. The item responses fell on a four-point scale, with responses including 1 (not been troubled at all), 2 (been a little troubled), 3 (been quite troubled), and 4 (been very much troubled). The mental health variable is an average score that is obtained from the mean of the 13 HSCL items in the 2007 survey (M = 1.60, Std = 0.57) and the mean of the 26 HSCL items in the 2015 survey (M = 1.66, Std = 0.56). Higher values indicate poorer mental health; however, the mean mental health score on the 2015 survey was 0.06 points higher than that on the 2007 survey, and the difference was statistically significant at the 0.01 level. The 2015 survey showed a significantly higher mean HSCL score than that of the 2007 survey when we compared the 13-item short version in both surveys.

### 2.5. Measures of Resilience

As a measure of resilience, we incorporated the 28-item Resilience Scale for Adolescents (READ) [29] in the questionnaire. Responses on this scale range from 1 (completely disagree) to 5 (completely agree) on a five-point Likert scale. The mean score of all 28 items from the READ scale was 3.95 in both surveys (Std = 0.59 in the 2007 survey and Std = 0.66 in the 2015 survey). Previous analyses on the READ scale identified five factors of resilience among Norwegian youths using the 2007 survey data [30]—namely, “family cohesion”, “personal competence”, “social competence”, “social resources”, and “structured style”. We used the five-factor structure to obtain five scores for resilience for each case by summing the items for each factor, taking the mean (minimum as 1 = completely disagree and maximum as 5 = completely agree), where a higher value indicated higher levels of resilience. However, by comparing the means of the five factors from the 2007 survey with those from the 2015 survey, we noticed some small and significant changes (see Table A2 in the Appendix A).

Overall, the READ scale has the same mean between the two surveys, but we observed increases in the factor means for structured style and family cohesion, and a decrease in the factor means for social competence and social resources.

### 2.6. Analysis Plan

All responses from both surveys were stored in IBM SPSS Version 25 (SPSS Inc., Chicago, IL, USA), which we also used to carry out our analyses. As our survey sampling procedure and data quality provided assurance of representativeness, we use unweighted data in our analyses. We first compared the prevalence of young people’s reporting of victimization across the four major forms of violence from the 2007 survey with those from the 2015 survey. Second, we compared the prevalence of mental health and resilience across the different victimization groups and across time. Third, we applied hierarchical regression analyses to first estimate the main effects of poly-victimization and resilience on the mental health of young people (Model 1), and to then detect whether resilience moderates the effect of poly-victimization on adolescents’ mental health by introducing interaction terms (Model 2). We include gender effects in both models, as gender is a persistent predictor of the dependent variable (mental health) in previous research [23,24].

## 3. Results

### 3.1. Prevalence of Poly-Victimization among Norwegian Youth: 2007 and 2015

There was a significant decrease in victimized youth from 2007 to 2015 when examining the different forms of violence in Norway; the exception to this was verbal abuse by peers, which increased by nearly 10 percentage points (see Table A1). Overall, there were significant changes in the prevalence with which young people in Norway reported being victims of violence. There was a nearly 10 percentage-point decrease in youths who witnessed domestic violence, an 8 percentage-point decrease in youths being victimized by physical abuse by their peers and parents, and a very small decrease in sexual abuse (from 21.3% in 2007 to 19.5% in 2015). However, verbal abuse is on the rise, especially among peers. Similarly, we saw a slight increase in youths who reported never experiencing any forms of abuse (from 33.2% in 2007 to 36.9% in 2015), while there was a significant decrease in the prevalence of poly-victimization (i.e., 22.5% of young people reported being victims of three to four forms of violence in 2007, while 19% of youths reported the same in 2015; see Table 1).

### 3.2. Background Variables, Mental Health, and Resilience among Youth Exposed to Multiple Forms of Violence

Table 2 presents the descriptions of the background variables that represented social disadvantages, mental health, and resilience among groups with different victimization profiles; here, the 2007 survey data are compared with the 2015 survey data. First, the group that reported being victims of multiple forms of violence was significantly different in terms of both gender and home background, particularly with respect to measures of mental health and resilience; this was true in both the 2007 and 2015 surveys. When examining the home background variables, participants who had identified as being poly-victims were significantly more likely to be female (2007: 63.4%; 2015: 67%) and from families that were from a poor economic situation (2007: 11.5%; 2015: 14%), while these participants were significantly less likely to be migrant youth (2007: 12.1%; 2015: 9%). The distributions of unemployed parents and parents without higher education remained stable in all groups between the two data-collection points. Although the changes from 2007 to 2015 are small, there was a significant increase in poor mental health among the groups that were victims of two or more forms of violence. While there was a small increase in resilience among young, non-victimized people, there was a significant decrease in resilience from 2007 to 2015 among young people who were victims of two, three, and four forms of violence. Our analyses of two youth populations from the two survey-administration periods indicated that, unfortunately, young people living with poly-victimization had poorer mental health and lower resilience than did those who were non-victims. We also observed another alarming trend: there was an increase in poor mental health and a decrease in resilience among young people, especially among those in the poly-victimization group.

### 3.3. Resilience as a Moderator of the Detrimental Effects of Poly-Victimization

Table 3 presents the hierarchical regression analyses that estimate the main effect of gender, poly-victimization, and resilience on the poor mental health of young people (Model 1), as well as the moderating effect of gender and resilience through the interaction terms of gender and poly-victimization, gender and resilience, and resilience and poly-victimization (Model 2). The results in Model 1 show that both gender and poly-victimization are positively associated with poor mental health, and these associations are similar across the two cohorts as well. Meanwhile, resilience has a strong, negative association with poor mental health and it is stronger for young people who participated in the 2015 survey (beta = 0.417) than for those who took the 2007 survey (beta = 0.384). With the introduction of three interaction terms in Model 2, there was a significant change in the R-squared value, with about 6% more variance explained in both the 2007 and 2015 data. A weak interaction effect of gender with poly-victimization was significant in 2007 alone (and not in 2015), which implies that gender only slightly moderates the relationship between poly-victimization and poor mental health. Meanwhile, the significant effect of the interaction between gender and resilience in both cohorts indicates that there was a moderating effect of gender on resilience, as related to poor mental health.

The results of Model 2 (presented in Table 3) met one of our study aims, which was to identify resilience as a moderator of the relationship between poly-victimization and poor mental health. The interaction of resilience with poly-victimization has a weak, negative, but significant association with poor mental health, which indicates that resilience moderates the relationship between poly-victimization and poor mental health. However, the moderating effects of both gender and resilience are very small; they appear to make no difference on the main effect of gender and little change to the strong main effects of poly-victimization either. Nevertheless, resilience seems to have a persistently stronger association with young people’s mental health than poly-victimization.

## 4. Discussion

In this study, we examined three phenomena: poly-victimization, mental health, and resilience, and the relationships between them. First, we explored the prevalence of poly-victimization among young people. Second, we examined the mental health and resilience of young people who completed two Norwegian national youth surveys. Finally, we identified resilience as a moderator of the detrimental relationship between poly-victimization and the mental health of young people. Our study identified those Norwegian adolescents who fit the definition for “poly-victims”, as they were victims of nearly all (three to four) forms of violence. A substantial proportion of the youth in our study reported being victims of poly-victimization in 2007 (22.5%) and in 2015 (19%). Our findings are similar to those reported elsewhere, such as in Sweden [14] and Canada [13].

Our results also show that the prevalence of poly-victimization has decreased slightly within the eight-year period encompassed by our study. The decrease in the prevalence of violence victimizations among children and youth is also observed in previous research (e.g., for physical violence [25], and sexual abuse [31]). However, these trends are inconclusive, as some population studies have noted that there are decreasing prevalence rates for some forms of violence, while others are increasing [25]. The reported decreases may be due to reductions in witnessing domestic violence, as well as reductions in violence perpetrated by parents (see Table A1), which was also observed in other studies [21]. Nevertheless, one of the most important findings from our study was the high prevalence of poly-victimization among Norwegian adolescents; this appears to be persistent across two youth cohorts that were surveyed eight years apart. However, a direct comparison is difficult to make given the lack of consensus of how to measure poly-victimization, as well as how to establish who can qualify as a poly-victim.

## 5. Conclusions

We observed a persistent, strong association between poly-victimization and the mental health of young people, which is in line with the findings from previous research [8,17]. Another important finding is that resilience has a strong and significant negative association with poor mental health, and it also substantially moderates the negative relationship between poly-victimization and young people’s mental health. The relationship between poly-victimization and poor mental health appears to be similar and stable in both surveys when accounting for the moderating effects of gender and resilience in the analysis. This indicates that resilience may protect an individual from developing poor mental health, especially among young people who are victims of multiple traumas. Our study offers useful insights for practitioners working with young people. Measures or strategies that are aimed at prevention or counselling initiatives that target young people who experienced violence should consider multiple approaches and cross-sectoral cooperation to foster and promote resilience; they should also target individual, family, and social dimensions.

However, we find it difficult to explain the other results related to resilience in our analyses, and these deserve further exploration in future research. Specifically, the following should be examined: (1) the significantly stronger association between resilience and mental health among those youth that participated in the 2015 survey when compared to those who completed the 2007 survey, and (2) the significant differences or changes in resilience factors between the two survey groups (see Table A2). While it has become apparent that resilience is increasingly more important in preventing mental health problems, there is a significant reduction in the two dimensions of resilience among youth (social competence and social resources), which necessitates further investigation.

Our study featured some limitations. Specifically, we were unable to estimate the prevalence rate of victimization; moreover, there were limitations associated with the representativeness of the Norwegian youth in this age group. As this was an upper-secondary school-based survey, the sample under-represented (or, unfortunately, excluded) two groups of young people. First, approximately 2% of Norwegian young people do not attend upper-secondary school upon completion of compulsory education. Second, approximately 30% of Norwegian upper-secondary school students drop out of school between the first and last years of their education [32].

The significant finding of our study was the potentially moderating effect of resilience, which was found to be significantly and negatively associated with both poly-victimization and poor mental health. The results of our study support the conception of resilience as a personal trait that can be developed and fostered as part of an intervention tool or strategy for children and youth, particularly when they face violent situations and experience poor mental health.

## Figures and Tables

**Table 1:** Lifetime prevalence of victimization of each and all four forms of violence among Norwegian youth (2007 and 2015); results are presented in percent.

Forms of Abuse and Victimization of Violence	2007 (*N* = 7033)	2015 (*N* = 4531)
Verbal abuse **	37.4	44
Witness of domestic violence **	39.1	29.8
Physical abuse **	40.6	32.3
Sexual abuse *	21.3	19.5
Non-victimization **	33.2	36.9
Victimization of a single form of violence	24.6	24.9
Victimization of two forms of violence	19.7	19.2
Victimization of three forms of violence **	15.6	13.5
Victimization of four forms of violence **	6.9	5.5

Note: Differences between 2007 and 2015: * *p* < 0.05: ** *p* < 0.01.

**Table 2:** Descriptive analyses of respondents’ background variables, social disadvantages, mental health, and Resilience Scale for Adolescents (READ) scores among youth groups without or with different victimization profiles.

Background Variables	All Respondents		Non-Victims		Victims of a Single Form of Violence		Victims of Two Forms of Violence		Victims of Three or Four Forms of Violence **	
	2007	2015	2007	2015	2007	2015	2007	2015	2007	2015
Female, %	58.5	59.2	56.9	55.5	59.1	60.5	54.8	57.1	63.4 *	67.0 *
Both parents were born outside of Norway, %	8.8 *	9.3 *	8.6	11.4	6.9	7.4	7.8	7.9	12.1 *	9.0 *
Both parents unemployed, %	5.2	5.0	4.4	4.4	4.0	4.1	5.7	3.7	7.3	8.6
Both parents without tertiary education, %	36.6 *	35.5 *	35.1	34.8	36.5	34.9	35.1	33.8	40.3	39.5
Family from a poor economic situation in the past 2 years, %	5.2	5.5	1.4	2.0	4.1	3.7	6.0	6.0	11.5 *	14.0 *
Parents are not together, %	33.7 *	35.7 *	23.2	28.4	31.6	33.3	37.9	38.2	47.8	49.2
Mental health HSCL, mean (std.)	1.60 * (0.57)	1.66 * (0.56)	1.38 * (0.42)	1.42 * (0.42)	1.56 * (0.51)	1.60 * (0.48)	1.65 * (0.55)	1.78 * (0.57)	1.92 * (0.66)	2.04 * (0.63)
Resilience READ scale, mean (std.)	3.96 (0.59)	3.95 (0.66)	4.13 * (0.50)	4.19 * (0.55)	3.97 (0.56)	3.98 (0.61)	3.89 * (0.61)	3.81 * (0.65)	3.73 * (0.63)	3.59 * (0.72)

Note: * The difference between 2007 to 2015 was significant at the 0.05 level. **The group was significantly different from all other groups across all variables at the 0.05 level. HSCL: Hopkins Symptom Checklist; READ: Resilience Scale for Adolescents.

**Table 3:** Standardized coefficients of poly-victimization and resilience regression on poor mental health (HSCL scale means).

Predicting Variables	Model 1		Model 2	
	2007	2015	2007	2015
Gender (male = 0. female = 1)	0.271 **	0.261 **	0.271 **	0.262 **
Poly-victimization (0 = non-victim; 4 = victim of four forms of violence)	0.256 **	0.263 **	0.246 **	0.253 **
Resilience	−0.384 **	−0.417 **	−0.377 **	−0.407 **
Interaction: Gender–Poly-victimization			0.025 *	0.012
Interaction: Gender–Resilience			−0.056 **	−0.040 *
Interaction: Resilience–Poly-victimization			−0.048 **	−0.054 **
R-squared ***	0.360	0.368	0.418	0.424

* *p* < 0.01: ** *p* < 0.001. *** R-squared changes between Model 1 and Model 2 are significant for both 2007 and 2015. HSCL: Hopkins Symptom Checklist.

## Appendix A.

**Table A1:** Measures and lifetime prevalence of violence and abuse in Norwegian youth, as based on surveys collected in 2007 (*N* = 7033) and 2015 (*N* = 4531).

Type of Violence	Category of Victimization	The Question: Has Any of the Following Ever Happened to You?	2007 Survey	2015 Survey
		Measurement Items	Number of Cases (%)	Number of Cases (%)
Non-physical violence	By peers	1. Been seriously bullied by peers 2. Been sexually harassed by peers 3. Been threatened with violence by peers	2110 (30.0)	1837 (40.5)
	By parents	1. Your mother/father has thrown/hit/kicked something during an argument with you 2. Your mother/father threatened you with violence during an argument with you	1085 (15.4)	490 (10.8)
Witness domestic violence	Parents being verbally abused	1. Seen or heard your mother/father being shouted at 2. Seen or heard your mother/father being insulted or humiliated 3. Seen or heard your mother/father being threatened with violence	2472 (35.1)	1127 (24.9)
	Parents being physically abused	1. Seen or heard your mother/father being pushed or heavily shaken 2. Seen or heard your mother/father being pinched or having their hair pulled 3. Seen or heard your mother/father being slapped with an open hand 4. Seen or heard your mother/father being hit with a fist 5. Seen or heard your mother/father being beaten with an object 6. Seen or heard your mother/father really being beaten up 7. Seen or heard your mother/father being treated with other forms of violence 8. Your mother/father has ever been injured as a result of violence (three items describing the prevalence of the injury)	828 (11.8)	357 (7.9)
	Sibling was physically abused by a parent	If you have a sibling (or siblings), you have seen or heard your mother/father using violence against your sibling(s)	750 (10.7)	409 (9.0)
Physical violence	By peers	1. Been beaten without visible injury by peers 2. Been injured by violence perpetrated by peers without needing doctoral treatment 3. Been injured by violence perpetrated by peers while needing medical treatment	1949 (27.7)	850 (18.8)
	By parents	1. Your mother/father has pushed or heavily shaken you during an argument with you 2. Your mother/father has pulled your hair or pinched you during an argument with you 3. Your mother/father has slapped you with an open hand during an argument with you 4. Your mother/father has hit you with a fist during an argument with you 5. Your mother/father has beaten you with an object during an argument with you 6. Your mother/father has really beat you up during an argument with you 7. Your mother/father has inflicted other violence on you during an argument with you 8. You have ever been injured as a result of violence by your mother/father (three items describing the prevalence of the injury)	1688 (24)	935 (20.6)
Sexual abuse	Unwanted sexual events (with physical contact)	1. Someone touched you inappropriately, against your will 2. You have touched someone else inappropriately, against your will 3. You have had intercourse against your will 4. You have had oral sex against your will 5. You have had anal sex against your will 6. You have had other forms of sex against your will 7. Someone has tried to rape you 8. You have been raped	1500 (21.3)	885 (19.5)

Note: Response alternatives and time of events were different in the surveys: (1) with respect to violence by peers, response alternatives were ‘yes’ or ‘no’ and the time of events was asked as “during or before the past 12 months” in the 2007 questionnaire, while in the 2015 questionnaire, response alternatives were ‘never’, ‘once’, ‘a few times’, ‘monthly’, ‘weekly’, or ‘daily’ and the past was asked as “before/after the age of 13”; (2) with respect to witnessing domestic violence, the response alternatives were ‘yes’ or ‘no’ and the time of events was asked as “during/before the past 12 months” in the 2007 questionnaire, while in the 2015 questionnaire, response alternatives were ‘never’, ‘once’, ‘a few times’, ‘monthly’, ‘weekly’, or ‘daily’, and the past was asked as “how old were you when it happened?”, with multiple-choice response options that included ‘under 5 years old’, ‘6–10 years old’, ‘11–13 years old’, and ’14 years and older’. (3) With respect to violence by parents, the response alternatives were ‘yes’ or ‘no’, and in the past it was asked as “before/after the age of 13” in the 2007 questionnaire, while in the 2015 questionnaire, response alternatives were ‘never’, ‘once’, ‘a few times’, ‘monthly’, ‘weekly’, or ‘daily’, while in the past it was asked as “how old were you when it happened?” with multiple-choice response options including ‘under 5 years old’, ‘6–10 years old’, ‘11–13 years old’, and ’14 years and older’. (4) Finally, with respect to unwanted sexual events, response alternatives were ‘yes’ or ‘no’ in the 2007 questionnaire; these were changed to ‘never’, ‘yes, once’, and ‘yes, several times’ in the 2015 questionnaire. In addition, the 2015 survey used a spilt-half method by which half of the respondents responded to the question that was formulated in the same way as in the 2007 survey, while half of the respondents responded to modified items that featured the words ‘against your will’ in each event. Previous analyses show no difference in prevalence rates between the spilt-half groups [25].

**Table A2:** Descriptive analyses of the READ scale: Item means and the five-factor structure for the 2007 and 2015 surveys.

Factors and Items of READ Scale	2007 Survey		2015 Survey	
	Mean	Std.	Mean	Std.
**Personal competence**	**3.79**	**0.72**	**3.78**	**0.79**
I will reach the goal if I persist **	4.5	0.8	4.4	0.9
I am satisfied with my life now **	4.2	1.0	4.0	1.1
I know how I will achieve my goal	3.9	1.0	3.9	1.1
When it is impossible for me to change things, I stop thinking about them	3.0	1.2	3.0	1.2
I feel competent **	3.7	1.0	3.7	1.1
When I make a choice, I often know which is the right one for me *	3.8	1.0	4.0	0.9
My faith in myself gets me through difficult times	3.6	1.2	3.6	1.2
When faced with adversity, I tend to find something good I can grow on	3.6	1.1	3.6	1.2
**Social competence ****	**3.99**	**0.74**	**3.91**	**0.82**
It is easy for others feel comfortable around me **	4.3	0.8	4.2	0.9
It is easy for me to make new friends **	3.9	1.1	3.7	1.2
I am good at talking to new people **	3.8	1.1	3.8	1.2
I always find something funny to talk about	3.8	1.0	3.8	1.0
I always find some comforting words to say to those who are sad	4.0	0.9	4.0	1.0
**Structured style ***	**3.40**	**0.79**	**3.47**	**0.84**
I work best when I have made clear goals **	4.1	0.9	4.1	0.9
I always make a plan before I start something new *	3.2	1.1	3.4	1.2
I am good at organizing my time *	3.1	1.2	3.2	1.2
In my family, we have rules that simplify everyday life *	3.1	1.1	3.2	1.2
**Family cohesion ***	**4.05**	**0.80**	**4.12**	**0.82**
In my family, we agree on what is important in life	4.0	1.1	4.0	1.1
I enjoy my time with my family	4.5	0.9	4.5	0.9
In my family, we agree on most things *	3.7	1.1	3.9	1.1
My family looks positively forward even when something sad happens *	4.1	0.9	4.2	0.9
In my family, we support each other	4.4	0.9	4.4	0.9
In my family, we like to do things together *	3.6	1.1	3.7	1.2
**Social resources ****	**4.48**	**0.60**	**4.39**	**0.69**
I have some friends/family members who tend to encourage me **	4.5	0.8	4.3	0.9
My friends always stick together **	4.3	0.9	4.2	1.0
I have some close friends/family members who really care about me **	4.8	0.6	4.7	0.7
I always have someone who can help me when I need it **	4.3	1.0	4.2	1.0
I have some close friends/family members who appreciate me **	4.5	0.8	4.5	0.8
Total resilience scale mean	3.96	0.59	3.95	0.66

* A mean increase is significant at the 0.05 level; ** a mean decrease is significant at the 0.05 level. Note: factors and their means and standardized deviations are written in bold to differentiate them from items.
